# Phenotypic and Methylome Responses to Salt Stress in *Arabidopsis thaliana* Natural Accessions

**DOI:** 10.3389/fpls.2022.841154

**Published:** 2022-03-04

**Authors:** Xiaohe Lin, Ming Zhou, Jing Yao, Qingshun Q. Li, Yuan-Ye Zhang

**Affiliations:** ^1^Key Laboratory of the Ministry of Education for Coastal and Wetland Ecosystems, College of the Environment and Ecology, Xiamen University, Xiamen, China; ^2^Graduate College of Biomedical Sciences, Western University of Health Sciences, Pomona, CA, United States

**Keywords:** accession-specific, *Arabidopsis thaliana*, genome-wide DNA methylation, salt stress, whole-genome bisulfite sequencing (WGBS)

## Abstract

Salt stress threatens plant growth, development and crop yields, and has become a critical global environmental issue. Increasing evidence has suggested that the epigenetic mechanism such as DNA methylation can mediate plant response to salt stress through transcriptional regulation and transposable element (TE) silencing. However, studies exploring genome-wide methylation dynamics under salt stress remain limited, in particular, for studies on multiple genotypes. Here, we adopted four natural accessions of the model species *Arabidopsis thaliana* and investigated the phenotypic and genome-wide methylation responses to salt stress through whole-genome bisulfite sequencing (WGBS). We found that salt stress significantly changed plant phenotypes, including plant height, rosette diameter, fruit number, and aboveground biomass, and the change in biomass tended to depend on accessions. Methylation analysis revealed that genome-wide methylation patterns depended primarily on accessions, and salt stress caused significant methylation changes in ∼ 0.1% cytosines over the genomes. About 33.5% of these salt-induced differential methylated cytosines (DMCs) were located to transposable elements (TEs). These salt-induced DMCs were mainly hypermethylated and accession-specific. TEs annotated to have DMCs (DMC-TEs) across accessions were found mostly belonged to the superfamily of *Gypsy*, a type II transposon, indicating a convergent DMC dynamic on TEs across different genetic backgrounds. Moreover, 8.0% of salt-induced DMCs were located in gene bodies and their proximal regulatory regions. These DMCs were also accession-specific, and genes annotated to have DMCs (DMC-genes) appeared to be more accession-specific than DMC-TEs. Intriguingly, both accession-specific DMC-genes and DMC-genes shared by multiple accessions were enriched in similar functions, including methylation, gene silencing, chemical homeostasis, polysaccharide catabolic process, and pathways relating to shifts between vegetative growth and reproduction. These results indicate that, across different genetic backgrounds, methylation changes may have convergent functions in post-transcriptional, physiological, and phenotypic modulation under salt stress. These convergent methylation dynamics across accession may be autonomous from genetic variation or due to convergent genetic changes, which requires further exploration. Our study provides a more comprehensive picture of genome-wide methylation dynamics under salt stress, and highlights the importance of exploring stress response mechanisms from diverse genetic backgrounds.

## Introduction

Excessive accumulation of water-soluble salts in the soil causes salinization, which negatively impacts plant growth and induces land degradation (van Zelm et al., 2020). Soil salinization is intensified in the era of climate change due to global warming, low precipitation, and sea-level rise (Munns and Gilliham, 2015). Increasing studies have investigated the morphological, physiological, transcriptional, and genetically based changes to understand plant response and adaptation to salt stress (Yin et al., 2010; Duan et al., 2013; Kim et al., 2013; Julkowska et al., 2016; Rahman et al., 2016; Miryeganeh et al., 2021). These studies have adopted non-model species, crops, and model species, including *Bruguiera gymnorhiza*, *Oryza sativa*, and *Arabidopsis thaliana*, and identified phenology and root architecture changes, physiological regulatory compounds of osmolytes and antioxidants, and genes and signal transduction pathways for the salt response. Furthermore, epigenetic modifications, such as DNA and histone modifications, have been considered to regulate gene expression and silence transposable elements (TEs), and are thus anticipated to play an essential role in the plant response and genome stability under salt stress (Yolcu et al., 2016; Yang and Guo, 2018; Chang et al., 2020; Wang et al., 2021). Exploring epigenetic changes under salt stress will provide critical insights into understanding the molecular mechanisms of salt response and adaptation.

Epigenetic mechanisms are generally considered to include DNA modifications, histone modifications, small interfering RNAs (siRNAs) and microRNAs, which affect chromatin structure, transcriptional accessibility, and genome stability (Cavalli and Heard, 2019). DNA methylation is a fundamental form of epigenetic modification and interacts with other epigenetic mechanisms. DNA methylation changes contribute to the regulation of plant growth, development, fruit maturation, and acclimation to biotic or abiotic stress (Candaele et al., 2014; Yamamuro et al., 2014; Zhang H. et al., 2018). In plants, DNA methylation mainly occurs in three sequence contexts: CG, CHG, and CHH contexts (H = A, T, or G). The methylation level in these sequence contexts varies among genomic regions (Zhong et al., 2013; Du et al., 2015; Zhang H. et al., 2018). For instance, constitutively expressed genes often present high levels of CG methylation on the gene bodies, but low levels of methylation close to transcriptional start and termination sites (Bewick et al., 2016; Bewick and Schmitz, 2017). In TEs regions, high methylation levels are usually observed for all three sequence contexts (Zhang et al., 2006).

Methylation changes in gene regions and TEs under stressful environments regulate gene expression and transposon activity, and potentially modulate plants’ stress response. Studies on *Arabidopsis thaliana* mutants *rdm16ros1* have found that decreased transcript levels of DNA polymerase V (PolV) cause loss of DNA methylation, which may relate to increased sensitivity to salt stress (Huang et al., 2013). In tetraploid rice (*Oryza sativa*), salt stress causes genome-wide CHH hypermethylation in the flanking regions of protein-coding genes and TEs (Wang et al., 2021). Similarly, global hypermethylation on TEs under salt conditions has also been revealed for mangrove species *Bruguiera gymnorhiza* (Miryeganeh et al., 2021). Furthermore, in *Triticum aestivum* L., hypermethylation of HKT genes in the coding regions associated with transcriptional downregulation appears to contribute to higher salt tolerance (Kumar et al., 2017). Nevertheless, these studies adopt only one or two specific genetic backgrounds, and the methylation dynamics of multiple genotypes, particularly natural accessions, remain unexplored. Investigating the DNA methylation dynamics of multiple natural accessions under salt stress will improve our knowledge of the response from natural populations composed of diverse genotypes.

The capacity to cope with stressful environments has been observed to vary across genotypes, often reported as genotype-by-environment (G × E) interactions (Sultan, 2000; Groot et al., 2017). Significant G × E interactions demonstrate that genotypes vary in response to environmental changes (Marais et al., 2013; Josephs, 2018). Genotypic variation in stress response has been traditionally observed in phenotypic and physiological responses, but is recently found in genome-wide transcriptional responses. In *Batrachochytrium dendrobatidis*, nine genotypes grown in three different temperatures show significant genotypic variations of temperature responses in zoospore and zoosporangium sizes and growth rate (Muletz-Wolz et al., 2019). Across three genotypes of *Andropogon gerardii*, researchers found strong evidence for genotypic variation in drought response of physiological traits, including maximum PSII efficiency, stomatal conductance, and instantaneous water use efficiency (Marais et al., 2013; Hoffman and Smith, 2021). Recently, transcriptional responses under different environmental perturbations have been reported to significantly vary across different genotypes in *Daphnia magna* (Orsini et al., 2018). These studies indicate that stress responses and the underlying molecular mechanisms could vary between different genetic backgrounds. Although previous large-scale studies have suggested that DNA methylation variation is controlled mainly by genetic variation (Dubin et al., 2015; Kawakatsu et al., 2016; Meng et al., 2016), studies under the framework of G × E interactions to explore DNA methylation changes in response to current environmental stress are still rare (but see Wang et al., 2016; Saban et al., 2020). Hence, it is emergent to explore DNA methylation changes from various genetic backgrounds to give a more comprehensive picture of epigenetic regulation under salt stress.

Here, we adopted four natural accessions of *Arabidopsis thaliana* origin from different geographic locations, and explored the phenotypic and methylome response under salt stress. We planted these accessions under the control and salt stress conditions, and measured the phenotypes for each plant. Leaf samples were collected from these plants and subjected to whole-genome bisulfite sequencing (WGBS) analysis. We asked the following specific questions: (1) do salt stress, accession and their interaction significantly affect plant phenotypes? (2) what is the general pattern of genome-wide methylation shaped by different environmental conditions and accessions? And (3) what are the divergent and convergent methylation changes across accessions relating to the epigenetic regulation under salt stress?

## Materials and Methods

### Plant Materials

*Arabidopsis thaliana* is a predominantly self-pollinating annual plant species. It originated from Africa, broadly distributed across Eurasia, and was introduced to North America around AD 1600 (Durvasula et al., 2017; Exposito-Alonso et al., 2018). Seeds collected from different geographic locations globally are preserved in the stock center and are commercially available (ABRC^1^). Due to historical evolutionary forces, seeds from different geographic locations, termed natural accessions, are genetically divergent (Alonso-Blanco et al., 2016). These features make *A. thaliana* an ideal plant to investigate the genetic variation underlying ecological responses, and *A. thaliana* is increasingly adopted in ecological and evolutionary studies (Pigliucci, 2002).

In this study, *A. thaliana* seeds were obtained from ABRC. We employed four natural accessions, Abd-0, Dja-1, Tol-0, and TRE-1, originally collected from geographical locations in the United Kingdom, Kyrgyzstan, United States, and France (Supplementary Table 1). Seeds were bulked in our growth room for two generations to eliminate parental or grandparental environmental effects induced by different origins or during seed storage.

### The Experimental Design

**FIGURE 1:**
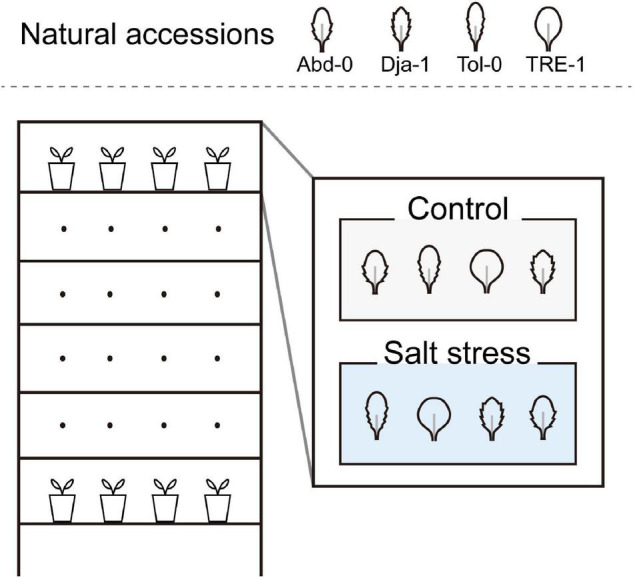
The experimental design. This experiment contains four natural accessions of *Arabidopsis thaliana* and two treatments of control and salt stress. The experiment follows a block design, and each block contains one plant from each natural accession. Two blocks compose a layer in the growth room and are assigned randomly to control and salt stress treatments. Six layers contain six replicated plants for each treatment and accession, and three replicated plants were sampled for whole-genome bisulfite sequencing (WGBS).

To explore the phenotypic response to salt stress with different genetic backgrounds, we planted the four accessions of *A. thaliana* in the growth room following a block design (Figure 1). Each block contained one plant per accession, and control and salt treatment were put into two separate blocks. The two blocks were replicated six times and distributed on the six layers of the shelf. Thus, this study contained six replicates per treatment per accession, amounting to 48 plants (=6 replicates × 2 treatments × 4 accessions). Accessions were randomly assigned among locations in a block, and the blocks were randomly assigned to the control or salt stress treatment.

Before germination, we put seeds in tubes with water and kept them at 4°C for a week. Less than ten seeds were grown on a pot (5.5 × 5.5 × 6 cm) containing a 1:1 mixture of soil and vermiculite. Within 10 days after sowing, we randomly thinned the seedlings to one plant per pot. For the control condition, we watered the plant every 4 days and added 50% Hoagland solution every 16 days. For the salt stress treatment, we watered the plants with 50 mmol / L salt solution every 4 days and 50% Hoagland solution every 16 days. About 55∼75 mL pure water or solution was given to a pot every time. We set the growth room condition to 16h:8h light/dark photoperiod, temperature between 22 and 24°C, and humidity between 60 and 70%. We considered the senescence of the plants as the sign to terminate the treatment and measure phenotypes, including plant height, rosette leaf diameter, fruit number, and aboveground biomass.

We measured the following phenotypes for each plant. Flowering time was observed daily and recorded as the days between germination and first blossom. We measured plant height as the length of the most extended stem/inflorescence. Rosette leaf diameter was measured as the longest diameter of rosette leaves. Fruit number was the total number of siliques counted on the harvested plants. Aboveground biomass was the dry weight after drying in an 80°C oven for 2 days. The reproductive allocation was calculated as the ratio of the fruit number to the aboveground biomass.

### Analysis of Phenotypic Data

To explore factors contributing to the significant phenotypic variation, we fitted the linear mixed effect model (LMM) to each trait. The model contained fixed effects of the layer, accession, treatment, accession-by-treatment interaction, and a random effect of the block. The model was formulated as *Y*∼*layer* + *accessions*×*treatment* + (1|*block*) with R version 4.1.0^2^, and analyzed with the function *lmer* in the package “lme4” (Bates et al., 2015). The significance of fixed effects was tested with *F*-test and Type II errors with the function *Anova* in the package “car” (Fox and Weisberg, 2019). The significance of the random effect was estimated with Chi-squared tests with the function *ranova* in the package “lmerTest” (Kuznetsova et al., 2017). To test whether the salt treatment significantly affect each accession, we fitted LMM formulated as *Y*∼*layer* + *accessions* + *accessions*:*treatment* + (1|*block*) to each phenotype, and adopted the function *summary* in the package “lmer4.”

### Genomic Variants Calling and Accession-Specific Reference Genomes

The TAIR10 reference genome of *A. thaliana*^3^ was assembled from the accession of Col-0, and there are genomic variants, including single nucleotide polymorphism (SNPs) and insertions and deletions (indels) between Col-0 and the accessions used in this study. Previous studies have suggested that these genomic variants would reduce the efficiency of data utilization and accuracy of mapping, especially when including samples with several different genetic backgrounds (Wulfridge et al., 2019). Furthermore, SNPs between samples and the reference, such as C-to-T and G-to-A SNPs, would cause false methylation estimation at related sites (Lea et al., 2017). To overcome these problems, we conducted whole-genome sequencing (WGS) for each accession and obtained accession-specific genomic variants. Through integrating the accession-specific genomic variants with the reference genome, we established four accession-specific reference genomes and used them as mapping references. In the accession-specific reference genomes, sites with C-to-T/G-to-A SNPs were updated to T/A, and were excluded from the subsequent methylation call.

We collected rosette leaves from one plant per accession and extracted DNA with the CTAB method. The sequence library was prepared with the Illumina TruSeq DNA library kit by Novogene (Beijing, China). Sequencing was conducted using Illumina NovaSeq 6000 System as 150 bp paired-end reads. We obtained an average of 39.2 million reads over samples (range: 28.3∼66.3 million), corresponding to 39–83 median depth after mapping (Supplementary Table 2). The sequencing quality of raw reads was assessed with FastQC v0.11.7^4^, and clean reads were obtained using Trimmomatic v0.36 (Bolger et al., 2014) with the parameters: LEADING:20 TRAILING:20 SLIDINGWINDOW: 4:20 MINLEN:50. Clean reads were aligned to *Arabidopsis* TAIR10 reference genome employing bwa v0.7.12 with default parameters (-T 30) (Li and Durbin, 2009). The generated alignment files were input to Picard v2.18.10^5^ to (1) create sequence dictionary (CreateSequenceDictionary); (2) order and sort bam files (ReorderSam, SortSam); (3) mark duplicates (MarkDuplicates); and (4) assign read group information including library, lane, and sample identity (AddOrReplaceReadGroups). Prior to calling genomic variants, we employed The Genome Analysis Toolkit (GATK, v4.0.8.1) to enhance alignment accuracy and correct base quality by implementing the following steps (McKenna et al., 2010). First, we used RealignerTargetCreator and IndelRealigner to detect and realign potentially erroneous indel sequences during alignments. Second, we used BaseRecalibrator to recalibrate the deviant base quality estimated by the sequencing machine.

After pre-processing of data, we implemented three steps to obtain accession-specific reference genomes. First, we adopted the GATK pipeline (v4.0.8.1) (McKenna et al., 2010) to identify accession-specific SNPs and indels. HaplotypeCaller parameters were set to –minimum-mapping-quality 40 -mbp 20 –filter-too-short 40 –max-fragment-length 600 -ERC GVCF -stand-call-conf 30. In the second step, we filtered the high-quality genomic variants with customized R scripts. The filtering criteria were set as follows: (1) the homozygous SNPs and indels were retained if the alternative allele supported by over 70% depth; (2) the depth of SNPs and indels should be between 3 and twice the median depth; and (3) following filter settings were applied separately: for SNPs, Quality by depth (QD) ≥ 20, RMS Mapping Quality (MQ) > 50, Fisher Strand (FS) ≤ 20, and Strand Odds Ratio (SOR) ≤ 3; for indels, Quality by depth (QD) ≥ 20, RMS Mapping Quality (MQ) > 50, Fisher Strand (FS) ≤ 60, and Strand Odds Ratio (SOR) ≤ 3. In the third step, we integrated the filtered genomic variants with the TAIR10 reference genome using BCFtools (Li, 2011) to obtain accession-specific reference genomes. In this step, only the homozygous SNPs and indels were included. As it changed ordinations in the genome, a chain file was generated to label these changes.

### Bisulfite Sequencing and Data Analysis

To investigate the methylome response under salt stress, we sampled three plants per treatment and accession, and 24 samples were analyzed. Two or three rosette leaves were collected from a plant before flowering and dried with silica gel at room temperature. We extracted DNA from dry leave materials with the CTAB method. We adopted the Whole-Genome Bisulfite Sequencing (WGBS) approach, which gives methylation level at single cytosine resolution. Sequencing library preparation was prepared with the Accel-NGS kit (Swift Biosciences), as it has a relatively low limit of DNA input (Luo et al., 2017) and unbiased coverage (Zhou et al., 2019). Libraries were sequenced with Illumina NovaSeq 6000 System to give 150 bp paired-end reads. Library preparation and sequencing were carried out by Novogene (Beijing, China). An average of 31.8 million reads were retained among samples (range:25.9∼37.5 million), corresponding to a median coverage of 34 × (range: 28∼43 ×) after quality control (Supplementary Table 2).

Sequencing quality was checked with FastQC v0.11.7 (see text footnote 4), and clean reads were obtained using Trimmomatic v0.36 (Bolger et al., 2014) with the parameters: LEADING:20 TRAILING:20 SLIDINGWINDOW: 4:20 MINLEN:50. We mapped the reads to the accession-specific reference genome using Bwa-meth v.0.2.2 (Pedersen et al., 2014) and default parameters. The mapping results were filtered by Samtools v.1.7 (Li et al., 2009) to remove reads below mapping quality 30. The mapped bam was deduplicated with Picard v2.25.2 (see text footnote 5). We extracted the methylation level for each cytosine using MethylDackel v.0.5.2^6^ with parameters -p 20 -q 40 --OT 0,0,0,135 --OB 0,0,5,0, and the methylation file was extracted for CG- CHG- and CHH-contexts. To correct ordination changes in accession-specific reference genomes, we adopted Liftover^7^ to covert accession-specific ordinations of methylation files to original TAIR10 ordinations.

The methylation level for a given cytosine was quantified as reads of unconverted C over total reads for this cytosine. To provide an overview of methylation differentiation between samples, we conducted PCA analysis for each context using the function *PCASamples* in R package “methylKit” (Akalin et al., 2012). We visualized the results using GGPlot2 (Wilkinson, 2011). To estimate the average methylation level of genes or TEs, we annotated the methylation file with TAIR10 annotations^8^ using function *findOverlaps* in the R package “GenomicRanges” v.1.42.0 (Lawrence et al., 2013) and plotted the results using the function *plot* in R basics.

For each genotype, we compared methylation profiles from the salt condition against those from the control condition, to explore the methylation dynamics under salt stress. In each condition, three methylation profiles were collected from three different biological replicates. We employed the R package “DSS” v.2.38.0 (Feng et al., 2014) to analyze differential methylated cytosines (DMCs) and regions (DMRs). The software implemented a general linear model with beta-binomial distribution to reveal DMCs, and clustered significant DMCs into DMRs (Feng et al., 2014). The test was performed on cytosines between coverage between 10 and twice the median depth, using the function *DMLtest* without smoothing. CG-DMCs were further filtered using function *callDML* with methylation difference ≥ 0.4, p.threshold = 0.05, and CHG and CHH-DMCs were filtered with methylation difference ≥ 0.2, p.threshold = 0.05. The filtration by depth and parameters was conducted following previous suggestions (Bewick et al., 2016; Bewick and Schmitz, 2017). The DMR analysis was performed based on DMC outputs using function *callDMR* with parameters p.threshold = 0.05.

We annotated salt-induced DMCs and non-DMCs to different genomic sites to explore the distribution of methylation changes. Non-DMCs were defined as the cytosines meeting the coverage standards (between 10 and twice the median depth) but were not tested to be DMCs. As the output of the DMC file did not contain “+/− strand” information, DMC and non-DMC profiles were merged with “strand” information from methylation calls produced by MethylDackel using the function *left_join* in the R package “dplyr.” We annotated DMC/non-DMC to TEs, exon, intron, 1 kb upstream of genes, 1 kb downstream of genes, and intergenic regions. To avoid assigning sites to overlapping genomic features, we annotated sites followed the order: TE > exon > intron > 1 kb upstream of genes > 1 kb downstream of genes > intergenic regions, where a site annotated to a former feature would be excluded from subsequent annotation. In this order, if a site was annotated to “1 kb downstream of genes,” it would not be annotated to the “intergenic region.” This annotation was conducted for different accessions, using customized scripts with R package GenomicRanges v.1.42.0 (Lawrence et al., 2013). We merged salt-induced DMCs across four accessions, and performed hierarchical clustering analysis with all CG- and CHG-DMCs, and 20,000 randomly selected CHH-DMCs, using the R package ‘‘pheatmap^9^.”

To explore the methylation dynamics on TEs, we extracted DMCs annotated to TEs. We used the DMC files with strand information to overlap with the “transposon_fragment” feature in TAIR 10. This annotation was conducted using customized scripts with R package GenomicRanges v.1.42.0 (Lawrence et al., 2013). If a TE was annotated with one or more DMCs, we referred to it as a DMC-TE in this study. With the annotation result for each accession, we adopted Venn diagrams to illustrate to what extent DMCs and DMC-TEs were exclusively found in one accession (accession-specific) or shared by multiple accessions, using the function *ggvenn* in the R package ‘‘ggvenn^10^.” Accession-specific and accession-shared DMC-TEs were classified into superfamilies and families with TAIR10 annotation.

To explore the methylation dynamics on genes, we also extracted DMCs and annotated them to genes and their proximal regulatory regions. The proximal regulatory region of a gene was defined as 1 kb upstream of transcription start site (TSS), using the function *flank* in GenomicRanges. This annotation was conducted using customized scripts with R package GenomicRanges v.1.42.0 (Lawrence et al., 2013). If one gene or its proximal regulatory region was annotated with one or more DMCs, we referred to it as a DMC-gene in this study. With annotation results for each accession, we adopted Venn diagrams to illustrate to what extent DMCs and DMC-genes were exclusively found in one accession (accession-specific) or shared by multiple accessions, using the function *ggvenn* in the R package “ggvenn.” We conducted Gene Ontology (GO) enrichment analysis on DMC-genes that were accession specific and shared by accessions, using the function *enrichGO* in the R package “clusterProfiler” v.3.18.1 (Yu et al., 2012). To reduce redundant enriched terms, significant enriched GO terms (*P* < 0.05) were input into REVIGO (Supek et al., 2011) and clustered according to semantic similarity with SimRel > 0.7. The output result list was filtered with dispensability < 0.20 to retain indispensable terms. The GO terms after reducing redundancy were visualized with the R package “GGPlot2” (Wilkinson, 2011).

Furthermore, we also investigated the methylation dynamics of DMRs on genes and TEs. The annotation and analysis procedure was similar to DMC and performed by customized scripts. A DMR was considered annotating to a genomic feature (a TE or a gene and its proximal regulatory region) if the DMR and genomic feature overlapped by one cytosine.

## Results

### Phenotypic Response to Salt Stress

**FIGURE 2:**
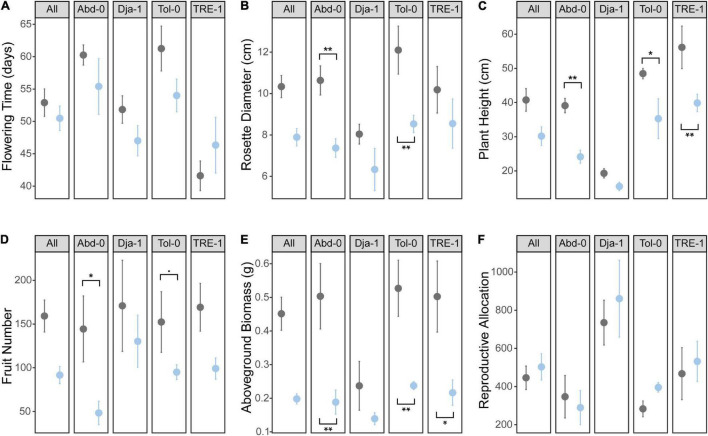
Treatment and accession effects on **(A)** flowering time, **(B)** rosette diameter, **(C)** plant height, **(D)** fruit number, **(E)** above-ground biomass, and **(F)** reproductive allocation. Asterisks indicate salt stress significantly changes the trait of an accession (“*” *P* < 0.05, “**” *P* < 0.01 and “.” *P* < 0.1). Black, control; blue, salt treatment.

The phenotypic results showed that salt stress significantly decreased plant height, rosette diameter, biomass, and fruit number of *A. thaliana* plants (Table 1 and Figure 2). Plants under salts tended to flower earlier than the control. Different accessions varied significantly for all phenotypes except fruit number (Table 1 and Figure 2). TRE-1 flowered earlier than other accessions, and Dja-1 was smaller in plant height, diameter, and biomass than others (Figure 2). Furthermore, we observed a marginally significant accession-by-treatment interaction for biomass, suggesting different accessions tended to have different responses to the salt stress. The response of Dja-1 in biomass appeared to be smaller in magnitude than other accessions (Table 1 and Figure 2).

**TABLE 1:** Summary statistics of linear mixed effect models.

			Flowering time		Rosette diameter		Plant height		Fruit number		Aboveground biomass		Reproductive allocation	
	df1	df2	F/Chi-sq	*P*	F/Chi-sq	*P*	F/Chi-sq	*P*	F/Chi-sq	*P*	F/Chi-sq	*P*	F/Chi-sq	*P*
**Fixed effects**														
Layer	5	5	0.51	0.760	0.55	0.739	0.66	0.671	0.12	0.984	1.25	0.406	0.63	0.686
Treatment (T)	1	5	1.03	0.359	17.77	**0.010**	23.61	**0.006**	7.53	**0.045**	14.68	**0.013**	0.20	0.676
Accession (A)	3	19–24	10.39	**<0.001**	4.95	**0.008**	38.32	**<0.001**	0.73	0.546	5.81	**0.004**	6.78	**0.002**
T × A	3	19–24	1.18	0.344	1.97	0.146	1.53	0.233	0.97	0.435	2.43	0.091	0.50	0.687
**Random effect**														
Block	1	–	1.02	0.313	<0.01	1.000	<0.01	1.000	<0.01	1.000	2.70	0.100	1.74	0.187

*Significant P values are given in bold.*

### An Overview of Methylation Variation

To explore the salt response at the molecular level, we conducted whole-genome bisulfite sequencing (WGBS). After quality control of raw reads, an average of 31.8 million reads were retained (range:25.9∼37.5 million), with an average of 89.1% uniquely mapped to the accession-specific reference genomes (87.2∼90.3%). The double-strand median depth ranged from 28 to 43 with an average of 34 across samples, and single-strand median depth ranged from 13 to 21, with an average of 16 (Supplementary Table 2). As this depth reached the recommended standards (Ziller et al., 2015), our results would provide a robust analysis of methylome response to salt stress at single cytosine resolution.

**FIGURE 3:**
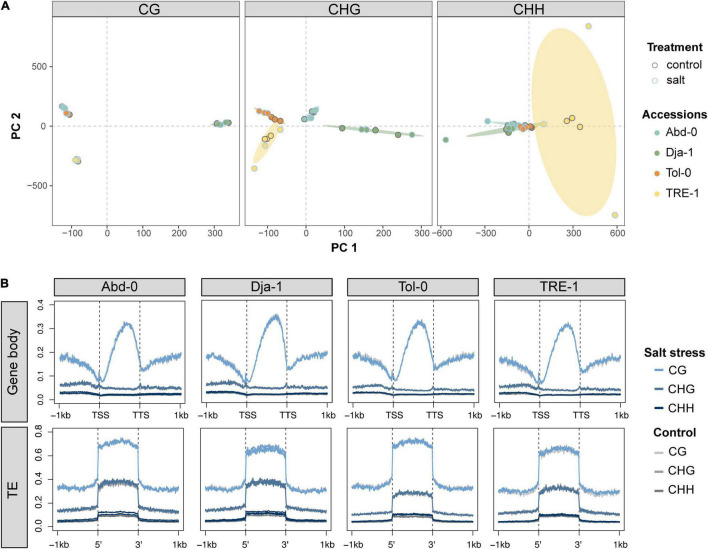
Primary component analysis results of genome-wide single cytosine methylation levels for each sequence context (CG, CHG, and CHH) **(A)**. Different filled colors indicate different accessions, and different edge colors indicate the control and salt stress conditions. **(B)** The averaged methylation levels over gene regions and transposable elements (TEs) under control and salt stress conditions.

PCA analysis of genome-wide single cytosine methylation showed that such methylation pattern was primarily clustered according to accessions, particularly in the CG and CHG contexts (Figure 3A). The average level of CG methylation on gene bodies and TEs also varied among accessions, with Dja-1 being slightly higher on gene bodies and Abd-0 slightly higher on TEs than other accessions. In general, salt stress appeared to have little effect on the average methylation level on gene bodies and TEs, but increased CHH methylation levels on TEs minorly (Figure 3B). These results suggest that, compared to the genetically based accession effect, the environmental effect of salt stress appeared to explain a minor part of the overall global methylation changes. This is possibly because hypermethylation and hypomethylation sites counteract, and the average methylation level remained unchanged under salt stress. Therefore, a detailed investigation of methylation dynamics at single-cytosine resolution is necessary.

**FIGURE 4:**
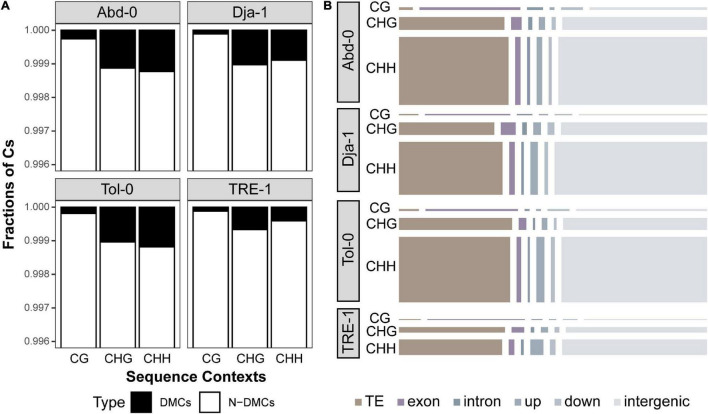
The percentage of differentially methylated cytosines (DMCs) caused by salt stress and non-differentially methylated cytosines (N-DMCs) across the genome for each accession **(A)**. The number of DMCs annotated to different genomic regions, including the TE, exon, intron, upstream and downstream of genes, and intergenic region **(B)**. The areas of the rectangles represent the number of (CG-, CHG-, or CHH-) DMCs annotated to the specific genomic region.

Then, we analyzed salt-induced methylation change at single cytosine resolution for each accession, using differential methylated cytosine (DMC) analysis. The results revealed a total of 29,909, 23,834, 28,319, and 7,734 DMCs for accessions of Abd-0, Dja-1, Tol-0, and TRE-1, representing 0.11, 0.08, 0.10, and 0.04% of cytosines over the genomes (Figure 4A). The DMCs for each accession were not equally distributed among contexts, and CHG- and CHH-DMCs composed a higher proportion to total DMCs than CG-DMCs (Figure 4A). When annotating these DMCs to different genomic regions, a substantial proportion of DMCs was located in intergenic regions. Besides intergenic regions, CG-DMCs were primarily found to occur on gene bodies, particularly exons, but CHG- and CHH-DMCs were more frequent to observe on TEs (Figure 4B; Supplementary Figure 1; Supplementary Table 3). Furthermore, CG- and CHG-DMCs were primarily clustered according to accessions, whereas CHH-DMCs tended to be clustered by different environmental treatments (Supplementary Figure 2). These results suggest that salt-induced CG- and CHG-DMCs are mostly accession-specific, but a substantial proportion of CHH-DMCs are potentially shared by different accessions. Besides DMCs, the analysis revealed 127, 109, 167, and 77 DMRs with an average length of 93.6, 132, 104, and 81.7 bp for Abd-0, Dja-1, Tol-0, and TRE-1 (Supplementary Figures 3A–B; Supplementary Table 4). These DMRs were annotated to 182 TEs and 196 genes and their proximate regulatory regions (1 kb upstream, Supplementary Figures 3C,D). However, the DMRs only included 3.95, 6.15, 8.29, and 12.9% of DMCs for different accessions, and represented a limited faction of methylation dynamics under salt stress. Therefore, we focused on the following DMCs analysis to give a more comprehensive picture of methylation dynamics over genomes.

### Salt Stress-Induced Differential Methylated Cytosines on Transposable Elements

**FIGURE 5:**
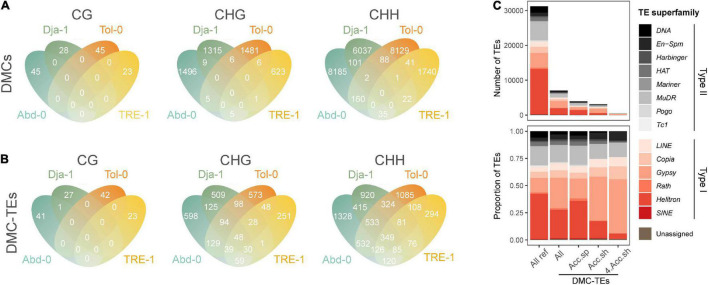
Methylation dynamics on transposable elements (TEs). The Venn diagrams of DMCs on TEs among accessions **(A)** and the Venn diagrams of TEs annotated with a DMC (DMC-TEs) among accessions **(B)**. DMCs in different sequence contexts (CG, CHG, and CHH) are annotated separately. **(C)** The number and proportion of all TEs in the reference genome, and all, accession-specific (Acc.sp), accession-shared (Acc.sh) and four-accession-shared (4 Acc.sh) DMC-TEs for different superfamilies. Acc. sp. refers to DMC-TEs found only in one specific accession, Acc.sh refers to DMC-TEs shared by at least two accessions, and 4 Acc.sh refers to DMC-TEs shared by all four analyzed accessions.

A total of 29,629 (33.5%) of total DMCs were annotated to TEs, including 141 CG-, 4,947 CHG-, 24,541 CHH-DMCs. Among these DMCs, an average of 89.2% were hypermethylated (increasing methylation level under salt stress), and 10.8% were hypomethylated (decreasing methylation level). The number of DMCs for different contexts is shown in Figure 5A for each accession. In general, these DMCs were accession specific, with 141 (100.0%), 4,913 (99.3%), and 24,091 (98.1%) of CG-, CHG-, and CHH-DMCs found exclusively in one accession (Figure 5A).

A total of 9,187 TEs were annotated to carry at least a CG or CHG or CHH-DMC (DMC-TEs), counting 26.4% of TEs in the reference genome. Compared to DMCs annotated to TEs that were primarily accession-specific (Figure 5A), a greater proportion of DMC-TEs were found in multiple accessions, with 1 (0.7%), 746 (27.9%), and 2,749 (43.2 %) TEs carrying CG-, CHG-, and CHH- DMCs found in at least two accessions (Figure 5B). For individual accessions, TEs carrying hypermethylated and hypomethylated DMCs were both overrepresented in the superfamily of *Gypsy*, a Type II transposon (Supplementary Figure 4). Intriguingly, the accession-specific DMC-TEs were relatively evenly distributed among families, but DMC-TEs found for multiple accessions were enriched in the *Gypsy* superfamily (Figure 5C). The *Gypsy* superfamily includes 32 families in the *A. thaliana* reference genome, and 29 of these families were found to have DMC-TEs in multiple accessions (Supplementary Figure 5). These results indicate that salt-induced DMCs repeatedly occur on a subset of TEs (mostly likely *Gypsy*) across natural accessions. Thus our study reveals relatively convergent salt-induced TE methylation dynamics across different genetic backgrounds.

### Salt Stress-Induced Differential Methylated Cytosines on Genes

**FIGURE 6:**
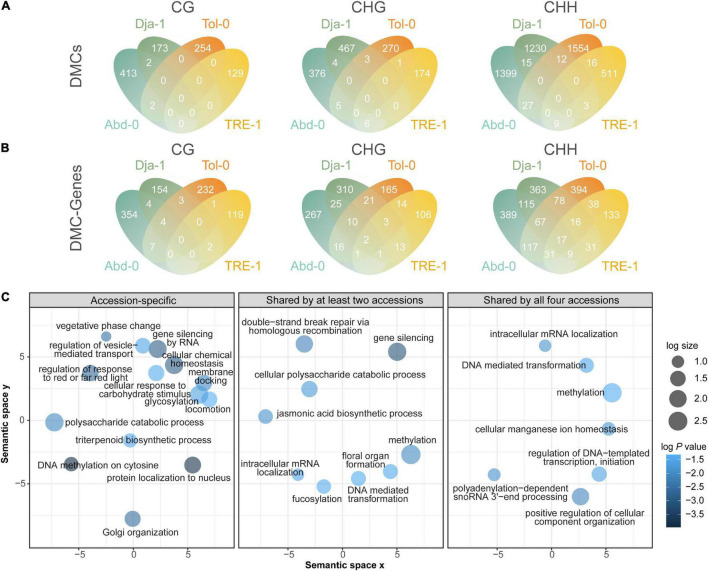
Methylation dynamics on genes and their approximate regulatory regions. Venn diagrams of DMCs on genes and the approximate regulatory regions among accessions **(A)** and DMC-genes among accessions **(B)**. **(C)** GO enrichment results of DMC-genes after reducing redundant terms. The GO enrichment analysis was conducted for accession-specific DMC-genes, DMC-genes shared by at least two accessions, and DMC-genes shared by all four analyzed accessions. The sizes of circles indicate the frequency of the significantly enriched GO terms (*P* values < 0.05) in the GO term database, where bubbles of more general terms are larger. The color of circles indicates the log10 transformed *P* values.

There were 7,055 (8.0%) of total DMCs annotated to gene bodies (the regions between the transcriptional start and termination sites) and their proximal regulatory regions (1 kb upstream of the genes). Among these DMCs, an average of 73.8% were hypermethylated, and 26.2% were hypomethylated. Most of these DMCs were also accession-specific, with 969 (99.6%) CG-, 1287 (98.5%) CHG-, and 4,694 (98.3%) CHH-DMCs exclusively found in one accession (Figure 6A).

A total of 3,090 genes or their regulatory regions were annotated to having one or more CG-, CHG, or CHH-DMCs. We referred these genes as DMC-genes, which accounted for 10.7% of genes in the reference genome. Compared to DMCs annotated to genes that were accession-specific (Figure 6A), a greater proportion of DMC-genes were found in multiple accessions, with 25 (2.8%), 123 (12.7%), and 561 (30.5%) genes carrying CG-, CHG-, and CHH-DMCs found in at least two accessions (Figure 6B). However, the proportion of DMC-genes found in multiple accessions was lower than DMC-TEs in the CHG and CHH contexts.

To explore the functions of DMCs on genes and regulatory regions, we conducted a Gene Ontology (GO) enrichment analysis of DMC-genes that were accession specific and shared by multiple accessions. The enrichment results reveal a rather convergence of pattern between accession-specific and accession-shared DMC-genes (Figure 6C). The convergent enrichment pathways were related to epigenetic and post-transcriptional regulations, including methylation and gene silencing (Figure 6C and Supplementary Figure 6). Furthermore, the convergent enrichment pathways were also associated with physiological and morphological salt responses, such as chemical homeostasis, polysaccharide catabolic process, and pathways relating to shifts between vegetative growth and reproduction. Given such dynamics reflecting their function in epigenetic regulation, our study indicates that, across different genetic backgrounds, methylation changes may have convergent functions in post-transgenerational, physiological, and phenotypic modulation under salt stress.

## Discussion

Taking advantage of different natural accessions of *A. thaliana*, this study investigated the effects of salt stress and different genetic backgrounds on plant phenotypes and genome-wide DNA methylation patterns. We found that genetic variations determined plant phenotypes, and salt stress also caused significant phenotypic changes. Whole-genome bisulfite sequencing (WGBS) revealed that gross methylation patterns were primarily determined by genetics, but salt stress caused significant methylation changes on about 0.1% cytosines over the genomes. Across different genetic backgrounds, these DMCs were repeatedly observed on the *Gypsy* superfamily of TEs and genes involving similar molecular functions. Our study thus provides a more comprehensive picture of DNA methylation dynamics under salt stress at a single cytosine resolution, and will provide insights into exploring the molecular mechanisms of salt response.

### Plant Phenotypes Were Determined by Genetic Background and Salt Stress

All measured phenotypes of *A. thaliana* significantly varied across natural accessions. Such genetically based variation between traits has been broadly observed for *A. thaliana* accession, and other ecologically relevant traits included morphology, physiology, and life-history traits (Atwell et al., 2010; Bergelson and Roux, 2010; Kover and Mott, 2012). The salt stress significantly reduced plant growth, and three of the four accessions were found to flower earlier under salt stress. Such phenotypic plasticity may be adaptive responses to buffer plants from environmental stresses, as these responses appear to increase the resource allocation to reproduction and ensure reproductive outputs (Kover and Mott, 2012; Freschet et al., 2018).

Furthermore, we also found a marginally significant accession-by-environment interaction, suggesting that salt stress-induced phenotypic plasticity tends to vary across accessions (Sultan, 2000; Uller, 2008). Consistent with previous work, phenotypic plasticity and resistance to salt stress of *A. thaliana* varied among accessions, albeit adopting different sets of accessions in the analysis (DeRose-Wilson and Gaut, 2011). Such accession-specific responses have also been reported in *A. thaliana* for various kinds of stresses, including heat (Groot et al., 2017), drought (Verslues and Juenger, 2011; El-Soda et al., 2015), light, and temperature (He et al., 2014), indicating divergent adaptive responses to stresses among natural accessions.

### The Overview of Genome-Wide Methylation Patterns

The genome-wide methylation patterns depended primarily on accessions, indicating genetic background is the primary determinant of methylation variation, which has been observed among the one thousand *A. thaliana* accessions (Kawakatsu et al., 2016), and the non-model species of *Plantago lanceolata* (Gaspar et al., 2019). Furthermore, methylation variation also occurs among recombinant inbred lines of *A. thaliana* (Zhang Y. Y. et al., 2018), *Glycine max L. merr.* (Schmitz et al., 2013a), and *Zea mays* (Eichten et al., 2013). Together these results show that methylation variation is frequently observed among different species and recombinant inbred lines, and a substantial proportion of DNA methylation variation depends on genetic variation.

Compared to genetic variation, we found that salt stress had a weak effect on the global methylation level on gene bodies and TEs. The minor changes in methylation level are consistent with the work of Annacondia et al. (2021), who found that aphid feeding induces no significant differences in global methylation levels across gene bodies and TEs. However, previous studies have revealed that nematode parasitism decreases methylation levels of susceptible lines of soybeans (Rambani et al., 2020). Heat stress has also been demonstrated to cause hypomethylation of the heat-sensitive line of cotton (Min et al., 2014). Different results between studies are possibly due to the various environmental stresses and genetic backgrounds. Therefore, it is critical to integrate diverse genetic backgrounds and environments and systematically analyze general changes of DNA methylation levels over the genomes.

Although there were slight changes in the gross methylation levels, we found pervasive methylation and demethylation dynamics at single cytosine resolution. To understand the methylation dynamics on TEs and genes, we employed the differential methylated cytosines (DMCs) and annotated DMCs to genomic regions. The importance of DMRs in regulating gene expression and phenotypes have been acknowledged by several previous studies (Dowen et al., 2012; Schmitz et al., 2013b; Ryu et al., 2018). Compared with these studies, our analysis identified a limited number (77–127) of salt-induced DMRs, covering (3.95–12.9%) DMCs across accessions. These results indicated that a large fraction of salt-induced methylation changes might be scattered across genomes, and focusing on the analysis of DMRs could ignore such part of methylation dynamics. Meanwhile, the sequencing depth of our study (28∼43×, Supplementary Table 2) appeared to give robust statistics of DMCs. Therefore, we put results of DMRs Supplementary and focus on DMCs in the main text, which will give a more comprehensive picture of DNA methylation dynamics under salt stress.

When annotating salt-induced DMCs to different genomic regions, the results showed that besides intergenic regions, CG-DMCs mainly occurred on exons, whereas CHG- and CHH-DMCs primarily occurred on TEs. This result is consistent with previous studies (Schmid et al., 2018; Annacondia et al., 2021), suggesting CGs are mainly responsibly regulating gene expression and CHG and CHH are involved in TE silencing. Clustering analysis of DMCs showed that CG- and CHG-DMCs were primarily clustered by accessions, and CHH-DMCs were firstly clustered by different environments (Supplementary Figure 2). A similar result was obtained in the populations of *Plantago lanceolata L.* (Saban et al., 2020). They found that while CG-, CHG- and CHH-DMCs all clustered according to the site of origins, only CHH-DMCs showed a clear separation between ambient and elevated CO_2_ conditions. These results indicated that CHH methylation changes might be more autonomous from genetic variation than other contexts, and under the regulation of environmental conditions. Testing the relationship between methylation and DNA sequence variation will elucidate the autonomy of methylation changes. Together, these findings suggest that methylation changes on gene bodies and TEs are associated with genetic backgrounds and environments. More details of methylome dynamics on genes and TEs are discussed in the following texts.

### Salt Stress Induced Methylome Dynamics on Transposable Elements

For methylome dynamics on TEs, most TEs annotated with one or more DMCs were hypermethylated, suggesting a potential suppression of TE activities in response to salt stress. Although most salt-stress induced DMCs were exclusive to one accession, these DMCs were found to occur repetitively on a subset of TEs across accessions. This subset of TEs mainly belongs to the superfamily of *Gypsy*. *Gypsy* is a superfamily of LTR retrotransposons, which composes 13.4% of TE copy numbers in the *A. thaliana* genome (Ahmed et al., 2011). Historically, plants’ genome size and organization during evolution have been proved to be closely associated with *Gypsy* (Vitte and Bennetzen, 2006). Furthermore, *Gypsy* is more prone to be activated than other superfamilies under stressful environments, including high salinity (Wang et al., 2015, 2018; Miryeganeh et al., 2021). Previous work observes that the heat stress activating TEs are significantly enriched in superfamilies of *Gypsy* and *Copia* in *A. thaliana* (Liu et al., 2021). Liu et al. (2021) also revealed that the methylation levels of *Gypsy* are increased under heat stress for H1 mutants. Consistent with the previous study, we found that the DMC dynamics are mostly the hypermethylation on *Gypsy*, suggesting that methylation changes in TEs under salt stress may involve inactivating TEs and stabilizing genome structures across different genetic backgrounds.

However, different DMCs responses in TEs were reported under alternative stresses. It was reported that aphid-induced methylation changes in TEs show significant enrichment in the *Rath* of *A. thaliana* genome (Annacondia et al., 2021). *Rath* is the most abundant superfamily of SINE, whose transpositions are highly dependent on the mechanisms of other retrotransposons (Quesneville, 2020). These differences between studies in TE methylation dynamics indicate that different stresses (i.e., biotic or abiotic) tend to affect TEs of different superfamilies. Joint analysis of TE activity and methylation dynamics will reveal the function of DNA methylation on TEs under stressful conditions.

### Salt Stress Induced Methylome Dynamics on Genes

We found that a greater proportion of DMC-genes were accession-specific than DMC-TEs, indicating that salt-induced methylation dynamics on genes depend on genetic backgrounds. This specificity of genetic backgrounds has been rarely explored among natural accessions, but was reported for lines used in breeding. In the study of nematode-infected soybeans, Rambani et al. (2020) found that resistant and susceptible lines have different sets of DMC-genes and our findings are generally consistent with this study.

Intriguingly, we found that accession-specific DMC-genes and DMC-genes shared by accessions were both enriched in similar GO terms of methylation and cellular chemical homeostasis. The epigenetic modification of methylation has been proposed to associate with plant response to various stressful conditions (Zhang H. et al., 2018). Furthermore, DMC-genes enriched in the function of methylation are also observed in the study of susceptible lines under nematode infections, which reported several differential methylated genes functioning in the process of DNA methylation (Rambani et al., 2020). Plants may activate the epigenetic regulation pathways, which cascadingly activates subsequent stress response pathways. Ionic stress is one of the major challenges for plants under high salinity (Zhao et al., 2020). The methylation changes in cellular chemical homeostasis pathways may alleviate the burden of the intracellular influx of high sodium ions. Together, the findings that accession-specific and accession-shared DMC-genes enriched in similar molecular functions indicate these molecular functions may be achieved through the epigenetic regulation of the same genes and of different genes with similar functions across accessions.

We also found that the DMC-genes shared by accessions were enriched in GO terms of the jasmonic acid (JA) biosynthetic process, polysaccharide catabolic process, floral organ formation, and vegetative phase change. JA plays critical roles in abiotic and biotic stress responses, including cold, drought, salinity, heavy metals, and light (Valenzuela et al., 2016; Yang et al., 2019; Wang et al., 2021). These DMC-genes may facilitate the biosynthesis of this plant hormone and activate the JA-signaling pathways. DMCs on genes involved in the polysaccharide catabolic process may mediate abscisic acid biosynthesis to cope with osmotic stress induced by high salinity (Zhao et al., 2021). Besides molecular and physiological responses, DMC-genes were also enriched in phenotypic responses, notably transition from vegetative growth to reproduction. This observation is consistent with the phenotypic changes we found in flowering time, which shows that plants tend to flower earlier under salt stress and increase reproductive allocation. As *A. thaliana* is an annual plant, earlier flowering is the adaptive life-history strategy to avoid stress conditions (Austen et al., 2017). These methylation dynamics on genes suggest that DNA methylation may be a critical molecular mechanism underlying the adaptive response to salt stress for *A. thaliana* natural accessions.

In summary, our studies provide a more comprehensive picture of phenotypic plasticity and DNA methylation dynamics under salt stress for different accessions in *A. thaliana*. Our study demonstrates that the phenotypic and methylome response are primarily determined by different genetic backgrounds. This finding is consistent with several largescale studies on the macro- and micro-evolutionary patterns (Schmitz et al., 2013b; Kawakatsu et al., 2016; Vidalis et al., 2016), suggesting that DNA methylation variation largely depends on genetic variation. Furthermore, our study focuses on the within-generational stress response and provides a detailed picture of methylome dynamics, suggesting that DNA methylation changes may stabilize the genome architecture and involve the epigenetic regulation of adaptive plasticity. Further investigation could survey the TE and gene expression to bridge epigenetic regulation and gene functions.

## Data Availability Statement

Whole-genome bisulfite sequencing data generated in this study have been deposited in the National Center for Biotechnology Information’s BioProject PRJNA790016. Scripts used to analyze methylation data are available at https://doi.org/10.5281/zenodo.5795425.

## Author Contributions

Y-YZ and XL designed the experiment. Y-YZ and QL supervised the study. XL, MZ, and JY carried out the experiments and collected the data. XL and MZ analyzed the data. XL wrote the first draft of this manuscript. All authors contributed to the revision of the manuscript.

## Conflict of Interest

The authors declare that the research was conducted in the absence of any commercial or financial relationships that could be construed as a potential conflict of interest.

## Publisher’s Note

All claims expressed in this article are solely those of the authors and do not necessarily represent those of their affiliated organizations, or those of the publisher, the editors and the reviewers. Any product that may be evaluated in this article, or claim that may be made by its manufacturer, is not guaranteed or endorsed by the publisher.

## Footnotes

1
https://abrc.osu.edu

2
www.r.project.org

3
https://www.arabidopsis.org

4
https://www.bioinformatics.babraham.ac.uk/projects/fastqc/

5
http://broadinstitute.github.io/picard

6
https://github.com/dpryan79/methyldackel

7
https://hgdownload.soe.ucsc.edu/goldenPath/hg19/liftOver/

8
https://www.arabidopsis.org/download_files/Genes/TAIR10_genome_release/TAIR10_gff3/TAIR10_GFF3_genes_transposons.gff

9
https://github.com/raivokolde/pheatmap

10
https://github.com/yanlinlin82/ggvenn

## Supplementary Material

Supplementary Figure 1 The proportion of DMCs and non-DMCs annotated to TEs, exon, intron, genes, upstream or downstream regulatory regions, and intergenic regions.

Supplementary Figure 2 Results of hierarchical clustering analysis with salt-induced DMCs.

Supplementary Figure 3 The number and proportion of DMC-TEs in different TE superfamilies. “Hyper-” indicates TEs annotated to have one or more hypermethylated DMCs under salt stress. “Hypo-” indicates TEs annotated to have one or more hypomethylated DMCs under salt stress.

Supplementary Figure 4 Boxplots for the number of cytosines within DMRs **(A)** and the length of DMRs for different accessions **(B)**. The Venn plots show the overlaps of DMR-TEs **(C)** and DMR-genes **(D)** among four accessions.

Supplementary Figure 5 The number of all TEs and DMC-TEs in different *Gypsy* families. Black bars indicate the number of all TEs in a *Gypsy* family summed from the reference genome. Different colored bars indicate the number of DMC-TEs in a *Gypsy* family summed for each accession.

Supplementary Figure 6 Gene Ontology (GO) enrichment results of DMC-genes after reducing redundant terms, shown for different accessions. The sizes of circles indicate the frequency of the significantly enriched GO terms (*P* values < 0.05) in the GO term database, where bubbles of more general terms are larger. The color of circles indicates the log10 transformed *P* values.
