# Supplementary figures and images for: Phenotypic and Methylome Responses to Salt Stress in Arabidopsis thaliana Natural Accessions
Source: Front Plant Sci. 2022 Mar 4;13:841154. doi: 10.3389/fpls.2022.841154 (PMC8931716; doi:10.3389/fpls.2022.841154)

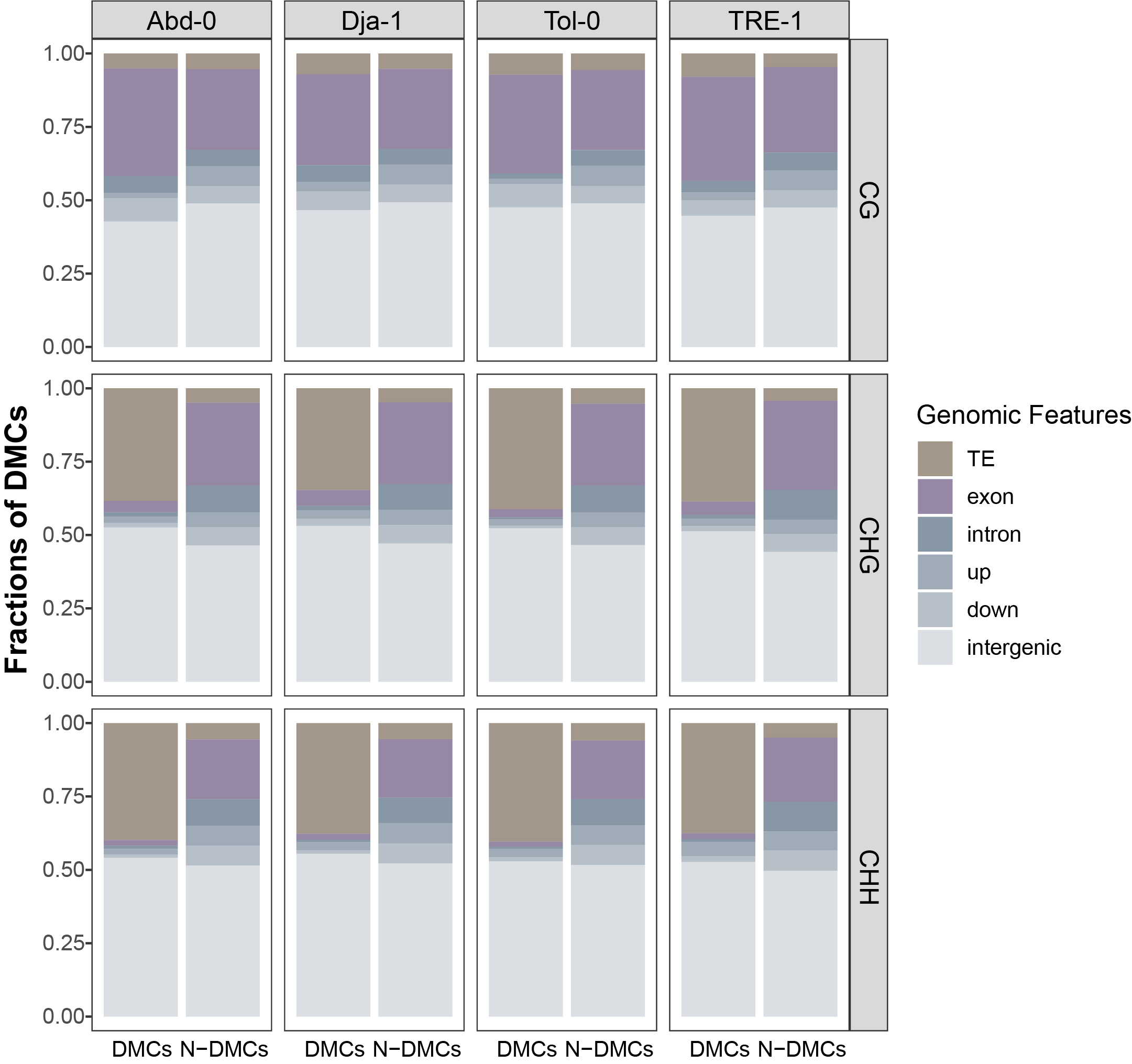

Supplement: Supplementary Figure 1 — The proportion of DMCs and non-DMCs annotated to TEs, exon, intron, genes, upstream or downstream regulatory regions, and intergenic regions. [file Image_1.jpg]

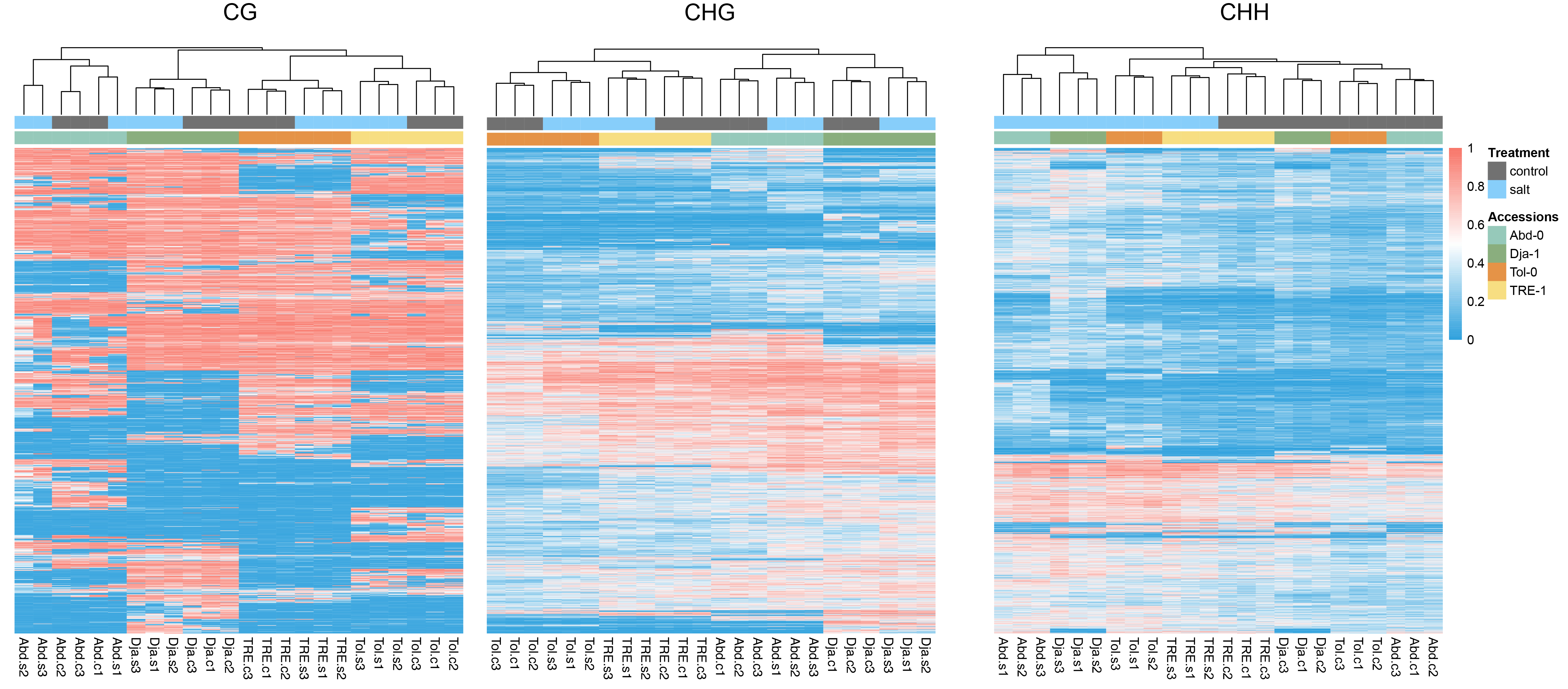

Supplement: Supplementary Figure 2 — Results of hierarchical clustering analysis with salt-induced DMCs. [file Image_2.jpg]

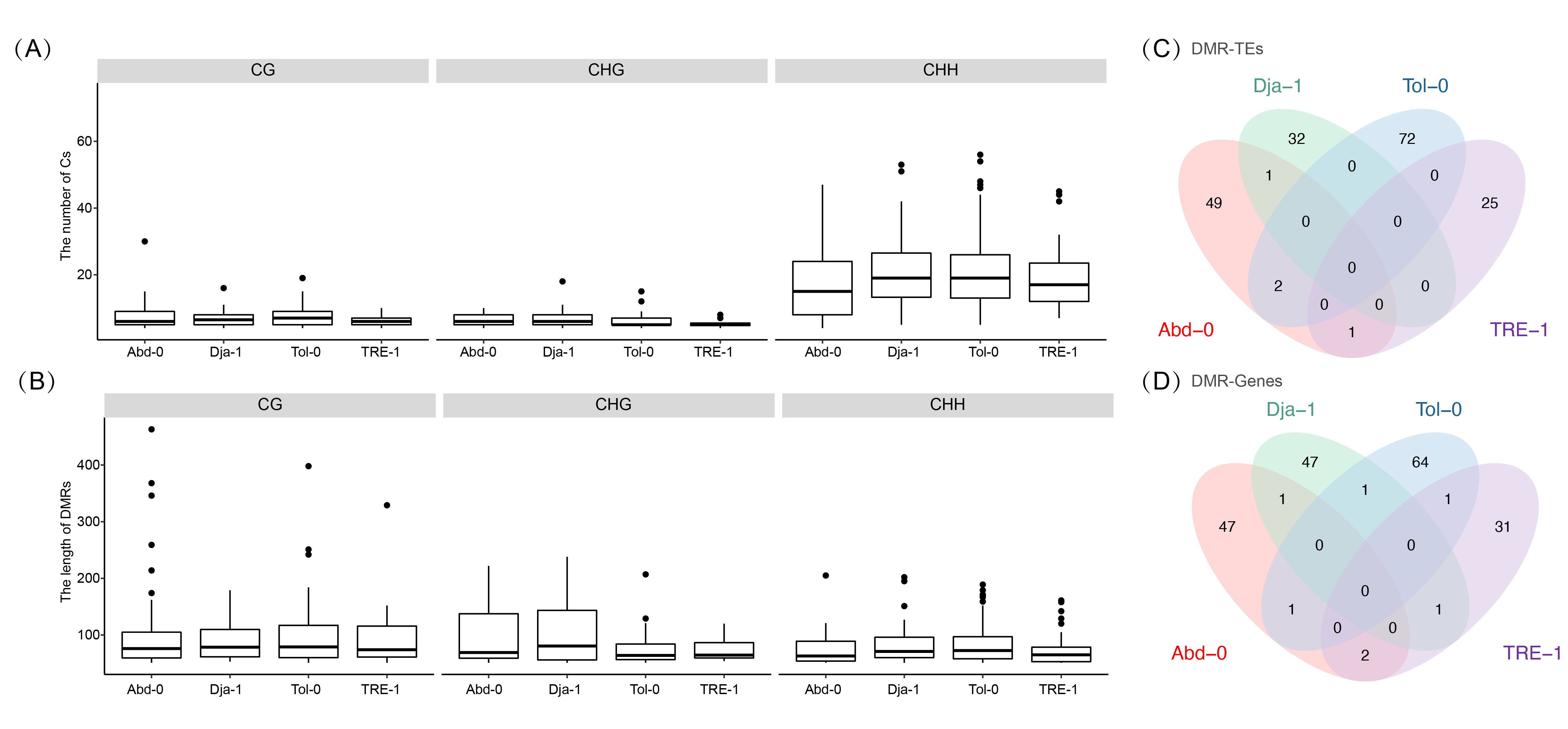

Supplement: Supplementary Figure 3 — The number and proportion of DMC-TEs in different TE superfamilies. “Hyper-” indicates TEs annotated to have one or more hypermethylated DMCs under salt stress. “Hypo-” indicates TEs annotated to have one or more hypomethylated DMCs under salt stress. [file Image_3.jpg]

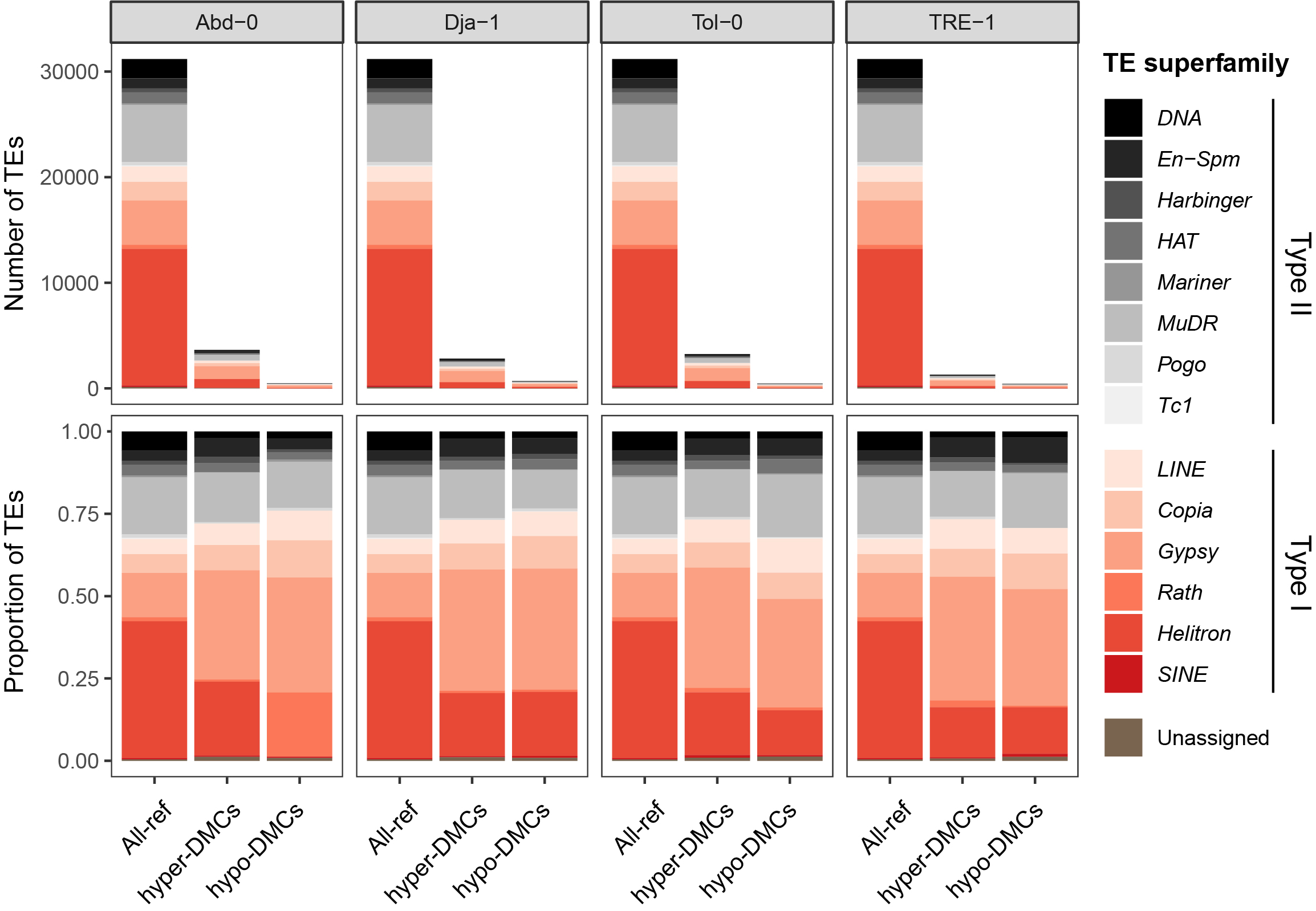

Supplement: Supplementary Figure 4 — Boxplots for the number of cytosines within DMRs (A) and the length of DMRs for different accessions (B). The Venn plots show the overlaps of DMR-TEs (C) and DMR-genes (D) among four accessions. [file Image_4.jpg]

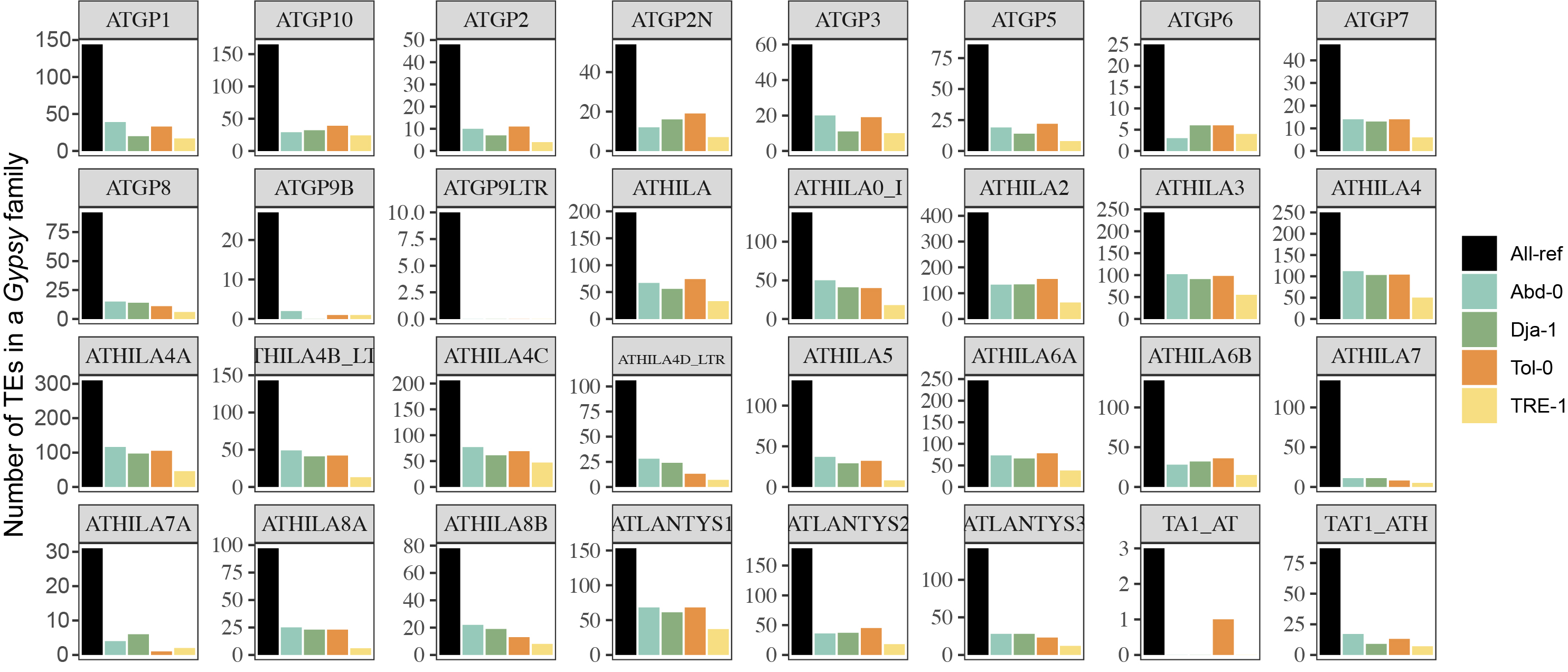

Supplement: Supplementary Figure 5 — The number of all TEs and DMC-TEs in different Gypsy families. Black bars indicate the number of all TEs in a Gypsy family summed from the reference genome. Different colored bars indicate the number of DMC-TEs in a Gypsy family summed for each accession. [file Image_5.jpg]

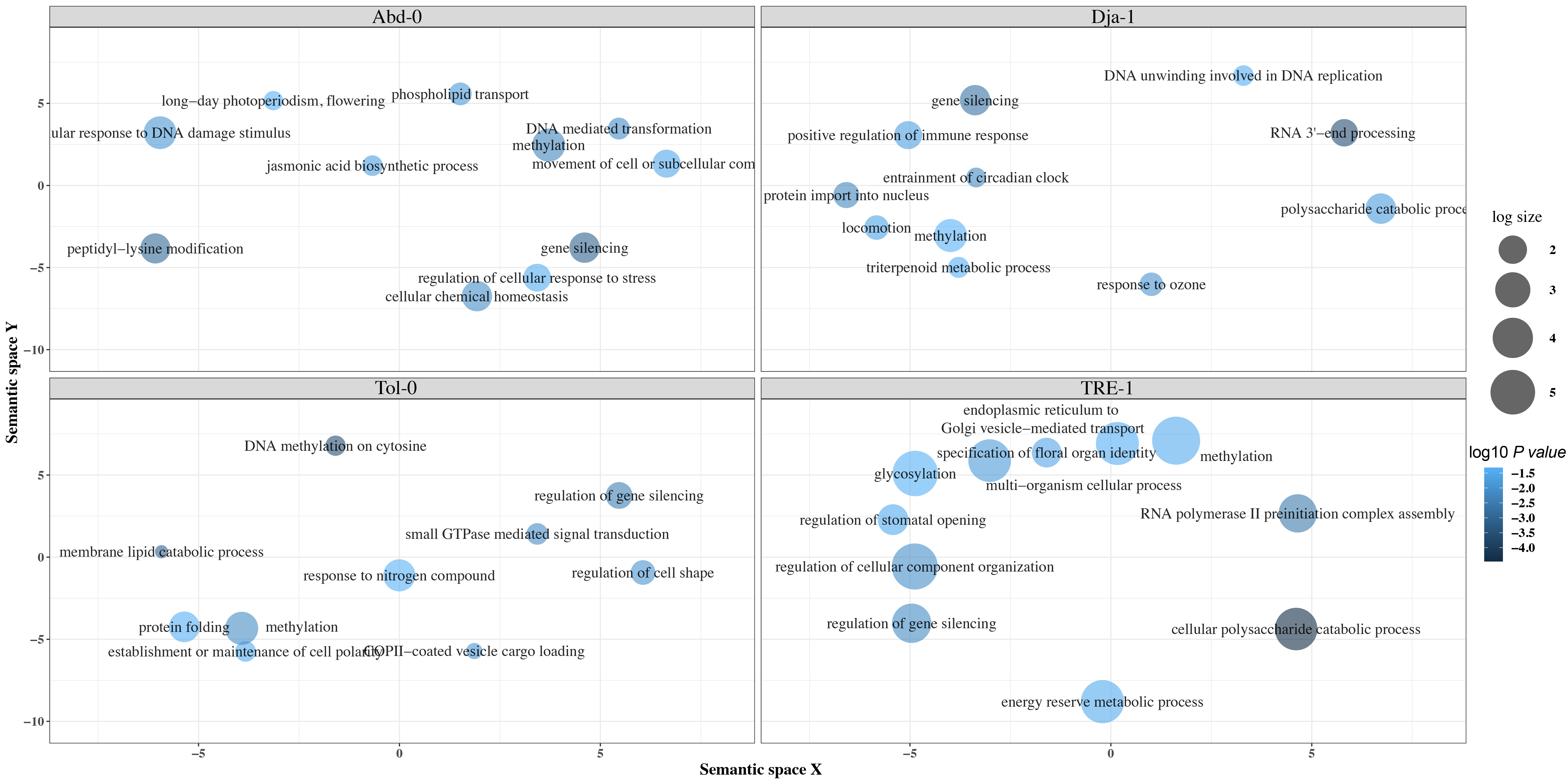

Supplement: Supplementary Figure 6 — Gene Ontology (GO) enrichment results of DMC-genes after reducing redundant terms, shown for different accessions. The sizes of circles indicate the frequency of the significantly enriched GO terms (P values < 0.05) in the GO term database, where bubbles of more general terms are larger. The color of circles indicates the log10 transformed P values. [file Image_6.jpg]
